# Stromal Fibroblasts in Tertiary Lymphoid Structures: A Novel Target in Chronic Inflammation

**DOI:** 10.3389/fimmu.2016.00477

**Published:** 2016-11-08

**Authors:** Francesca Barone, David H. Gardner, Saba Nayar, Nathalie Steinthal, Christopher D. Buckley, Sanjiv A. Luther

**Affiliations:** ^1^Rheumatology Research Group, Institute of Inflammation and Ageing, University of Birmingham, Birmingham, UK; ^2^Department of Biochemistry, Center for Immunity and Infection, University of Lausanne, Lausanne, Switzerland

**Keywords:** tertiary lymphoid structures, chemokines, tumor necrosis factor-alpha, lymphotoxin alpha1, beta2 heterotrimer, fibroblasts

## Abstract

Tertiary lymphoid structures (TLS) are organized aggregates of lymphocytes, myeloid, and stromal cells that provide ectopic hubs for acquired immune responses. TLS share phenotypical and functional features with secondary lymphoid organs (SLO); however, they require persistent inflammatory signals to arise and are often observed at target sites of autoimmune disease, chronic infection, cancer, and organ transplantation. Over the past 10 years, important progress has been made in our understanding of the role of stromal fibroblasts in SLO development, organization, and function. A complex and stereotyped series of events regulate fibroblast differentiation from embryonic life in SLOs to lymphoid organ architecture observed in adults. In contrast, TLS-associated fibroblasts differentiate from postnatal, locally activated mesenchyme, predominantly in settings of inflammation and persistent antigen presentation. Therefore, there are critical differences in the cellular and molecular requirements that regulate SLO versus TLS development that ultimately impact on stromal and hematopoietic cell function. These differences may contribute to the pathogenic nature of TLS in the context of chronic inflammation and malignant transformation and offer a window of opportunity for therapeutic interventions in TLS associated pathologies.

## Introduction

Organs are defined as collection of cells, extracellular structures, and fluids, joined into an operational unit to serve a common function. The anatomy of an organ is designed by its structural elements, or resident stromal cells, which provide shape and compartmentalization to the tissues. Secondary lymphoid organs (SLOs), which include spleen, lymph nodes (LN), and Peyer’s patches (PP), largely conform to this. SLOs hold a network of fibroblasts, vessels, and nerves that support a large mobile population of leukocyte and which support immune surveillance and response to noxious agents (1, 2). This elegant organization of SLOs develops in a highly conserved and regulated process that largely occurs, both in mice and humans, in embryonic and early postnatal life (3, 4). SLOs evolved simultaneously with the development of an adaptive immune system in vertebrates, with hundreds of LN being distributed at strategic sites, thereby providing a platform for immune cell clustering at well-defined areas. This enables rapid and more efficient adaptive immune responses that outpace pathogen replication, spread, and pathology (5).

Well-developed tertiary or ectopic lymphoid structures (TLS or ELS) resemble SLOs anatomically, as complex aggregates of leukocytes and specialized stromal cells. However, TLS are not capsulated and lack an independent vascular network. TLS form within non-lymphoid tissue in response to specific pathogenic events (6–8) and are commonly found, in adult life, at sites of chronic inflammation and cancer (9–12). It is likely that the capacity to form TLS preceded the development of SLO during evolution, as a tool to accumulate innate immune cells at sites of inflammation in non-vertebrates and lower vertebrates, such as birds, amphibians, and reptiles (13, 14).

Mucosa-associated lymphoid tissues (MALT), such as cryptopatches (CP), fat-associated lymphoid clusters (FALC), and induced nasopharynx-associated lymphoid tissue (iNALT), may be placed, both anatomically and developmentally, between SLOs and TLSs. These are pre-programed in time and space, developing either pre- or postnatally at predetermined sites but are able to expand and accommodate specialized immune responses if required. In CPs, their development into IgA plasma cell-rich isolated lymphoid follicles (ILF) is a classic example of this phenomenon (5). Interestingly, ILF are reversible structures, as indicated by their disaggregation upon antibiotic treatment (15, 16). Similarly, TLS are considered reversible once the antigen source and inflammatory signals are cleared, as will be discussed later.

TLS anatomy is plastic and highly variable, as is its cellular composition. While a certain degree of T/B cell segregation, vascular specialization, and lymphoid tissue chemokine expression is often observed, the level of organization and formation of germinal centers (GC) is dependent on the context, stage, and site of the immune response. Unraveling the mechanisms responsible for the differential maturation of the stromal and leukocyte compartments in TLS and the functional differences between SLO and TLS has provided key information on the biology, clinical relevance, and role of those structures as potential therapeutic targets. In this review, we discuss in detail the contribution of stromal cells, most notably fibroblasts, to both SLO and TLS development and function, and the potential to target therapeutically this specific cell type.

In a “resting state,” all organs of the body contain fibroblasts that provide structure and mechanical strength to the tissue. Fibroblast phenotype and function greatly differ between various anatomical sites, as shown by the extensive transcriptional differences detected in fibroblasts isolated from different compartments in diverse locations of the body (17). This specialization is further enhanced by the specific ability of fibroblasts to respond to a series of cytokines and inflammatory stimuli, which increase their proliferative capacity and induce functional adaptations to the environment (18–22). This review will focus on a subset of fibroblasts defined as “lymphoid tissue fibroblasts,” which inhabit SLOs and TLS, are characterized by extensive plasticity and specialized functions, and have recently emerged as important regulators of adaptive immunity (8, 23–25).

## Lymphoid Stromal Cell Differentiation in SLOs and TLS

In order to understand the TLS development, it is useful to review the development of LN and PP, which is initiated in the sterile environment of the embryo, at approximately E11 and E15, respectively. The locations at which LN develop are predetermined, at least in part, by endothelial expression of the lymphotoxin β receptor (LTβR) (26). Activation of the LTβR signaling pathway enables the clustering of CD45^+^CD4^+^CD3^−^ hematopoietic lymphoid tissue inducer (LTi), also known as type 3 innate lymphoid cells (ILC3) expressing LTα1β2, TRANCE, and RORγt (3, 4).

The origin and identity of the signals that induce specification of the mesenchymal progenitor cells prior to LTi arrival remain largely unknown. It is clear that a close anatomical and functional connection between immune cells, mesenchyme, and vascular structures is critical for the establishment of the anlage. At around E13.5, it is possible to identify the nascent LN anlage as small clusters of endothelial cells expressing both podoplanin (gp38) and ICAM-1. The anlage are surrounded by a layer of mesenchymal cells expressing PDGFRα, fibronectin, and ER-TR7 and is separated from the outer layers of fibroblastic cells by a thin Perlecan^+^ basement membrane (27, 28). The early differentiation of the mesenchyme and the initial upregulation of ICAM-1 and VCAM-1, CXCL13 and IL-7, occurs at this stage in the absence of LTα1β2 or LTi cells and is thought to be regulated by retinoic acid signals released by neurons and/or ILC3 (29, 30). Nonetheless, LTα1β2 is required for the further upregulation of adhesion molecules and of lymphoid chemokines. LTα1β2 binds specifically to LTβR and activates the alternative pathway of the NF-κB cascade, while TNFα and LTα3 have TNFR1 and TNFR2 as main receptors and activate the canonical NF-κB pathway (1, 3). The combined activation of TNFR1- and LTβR-signaling pathways not only leads to the activation of both the classical and alternative NF-κB pathways, which act in synergy but also shows complex cross-regulation. In addition, LTβR can be also activated by LIGHT (31). LTβ-receptor regulates in fibroblasts and endothelial cells the expression of various chemokines (CCL19/21, CXCL13) and survival factors (BAFF), while TNF receptor is required for the production of adhesion molecules, such as VCAM-1, ICAM-1, and MAdCAM (32–35). The activation of the alternative pathway, demonstrated by the expression of NIK and the transcription factor RelB, is a specific requirement for the activated mesenchyme to mature in a lymphoid tissue organizer cell (LTo) (1–3). Mature LTo specification facilitates, in turn, further attraction and retention of more LTi cells through the binding of CXCR5, CCR7, α4β1, α4β7, and LTα1β2 with their respective ligands (2, 3). These later steps coincide with the ingrowth of the mesenchymal layer into the endothelial bud (27, 28, 36, 37). The physical interaction between hematopoietic LTi and stromal LTo cells establishes a positive feedback loop that reinforces the formation of the cluster and leads to the stabilization of the anlagen with vascular differentiation, development of high endothelial venules (HEVs), and eventually attraction and compartmentalization of mature lymphoid and myeloid cells (2–4).

Utilizing a CCL19-Cre dependent LTβR ablation (*Ccl19-Cre* × *Ltbr^fl/fl^* mice), Ludewig and colleagues have recently shown that CCL19^+^ myofibroblastic stromal cell precursor cells can develop the basic LN infrastructure even in absence of LTβR triggering (38). Nonetheless, fibroblastic LTo cells require LTβR signaling to reach full maturation and immunological competence that includes strong expression of ICAM-1, VCAM-1, CCL19, CCL21, IL-7, and RANKL (28, 38, 39). Of note, LTo responsible for the aggregation of different lymphoid tissues are not uniform. This is suggested by the observation that embryonic LTo cells in PP, mesenteric, and peripheral LN display transcriptional differences as well as differential cellular and molecular requirements (40, 41).

Interestingly, LN development is associated with but not fully dependent on a functional lymphatic vasculature network. As a consequence, embryos lacking the major transcriptional regulator for lymphatic cell development, Prox1, either due to full or conditional *Prox1* deletion, fail to form mature LN. Both mutants develop hypoplastic LN anlagen containing small LTi clusters in areas of activated mesenchyme (42). Similarly, Clec-2 knockout mice, which exhibit a defect in lymphatic endothelial cell proliferation late in embryogenesis, form hypoplastic LNs with a mixture of blood and lymphatic flow and reduced LTi and LTo numbers (43).

Evolutionarily more ancient than LNs is the spleen that, together with gut-associated lymphoid tissue (GALT), represents the oldest SLO. The spleen is present in bony fish, amphibians, and reptiles, although in a less complex organization than that observed in mammals (14, 44). The development of the splenic white pulp cords that starts at birth in mice (45–48) and after 15 weeks of gestation in humans (49) does not require LTi cells or LTα1β2 (14, 44, 50, 51). However, as observed in the LN, stromal cell maturation, chemokine expression, and lymphocyte compartmentalization still require LTα1β2 and TNFα (1, 3, 52–56). Those ligands are likely to be provided by B cells and, as a consequence, B cell-deficient mice display smaller spleens, with poorly developed T zones (47). In conclusion, spleen and LN development depend on different types of inducer cells but show a similar hematopoietic–mesenchymal cell interaction, which eventually leads to a similar pathway of fibroblast maturation and lymphoid tissue compartmentalization.

Lymph nodes and PP anlagen formation in the embryo resemble a “sterile inflammation” (5, 13) aimed at forming organs before and independently from the encounter of danger signals. Thereby, these organs collate in a single, highly organized space antigen-presenting cells, naïve lymphocytes, and stromal cells that enable the rapid generation of adaptive immune responses against pathogens.

Tertiary lymphoid structures formation in the adult shares many similarities with SLO development; however, the order of events and molecular mechanisms responsible for TLS development are significantly different from those regulating LN development and partially different from those of the spleen. First, TLS form in the presence of lymphocytes that are absent during embryonic SLO formation. Second, TLS do not develop as separate encapsulated organs but arise as part of highly inflamed tissues, in response to a requirement for lymphocytes to cluster, survive, and generate local, efficient antigen-driven responses. Activation of the resident vascular structures including the upregulation of homing molecules to enable lymphocyte recruitment is therefore a prerequisite of TLS assembly (7, 8). However, while influenced by increased recruitment and defective lymphatic drainage of leukocytes, TLS formation is not simply determined by retention of activated cells in the tissue (57).

Modification of tissue-resident stromal cells into functional lymphoid tissue-like fibroblasts represents another hallmark feature of TLS, specifically, the ectopic and largely segregated expression of chemokines, such as CXCL13, CCL21, CCL19, and CXCL12, and of lymphocyte survival factors, such as IL-7, BAFF, and APRIL (7, 8). Once fully matured, TLS can display a specialized network of follicular dendritic cells (FDCs) capable of driving a functional GC response, and HEVs, differentiated from perivenular capillaries (57). Local upregulation of PNAd, MAdCAM, ICAM-1, and CCL21, first on flat, and later on high endothelial cells, enables the recirculation through the inflamed tissue of naïve T and B lymphocytes, previously excluded by the absence of cognate ligands for CCR7 and L-selectin (8, 57–59).

As opposed to SLO that arise on the backbone of a poorly differentiated mesenchyme, TLS form within organs whose mesenchyme is postnatal and specialized to support local anatomical and functional requirements (8). Accordingly, TLS assembly relies on a larger variety of mesenchymal organizer cells, hematopoietic inducer cells, and cytokines (6–8, 60). TLS preferentially arise in close proximity to vascular or epithelial ductal structures adjacent to pericytes, smooth muscle cells, or myofibroblast-like cells that share many functional and phenotypic features of LTo cells. For example, leukocyte aggregates in the synovium of patients with rheumatoid arthritis (RA) form in close proximity to networks of αSMA^+^ fibroblastic cells (61), and LTβR^+^ aortic smooth muscle cells have been observed in vascular TLS that form in murine models of atherosclerosis (62). Focal lymphocytic aggregations that define Sjogren’s syndrome (SS) assume a classical periductal structure in close contact to a layer of gp38^+^ myofibroblastic or pericytic cells that define the ductal basal membrane (63). Similarly, fibroblastic reticular stromal cells that share LTo and lymphoid tissue features have been identified in several murine and human TLS, including SS, primary biliary cirrhosis, insulitis, RA, and lungs infected by *Pseudomonas aeruginosa* (Table 1). More recently, a marked increase in the frequency of PDGFRα^+^PDGFRβ^+^Cadherin-11^+^ICAM-1^+^ fibroblasts has also been identified within cerebral lesions of mice that develop experimental autoimmune encephalomyelitis (EAE) (64). Recently, endothelial cells and perivascular fibroblasts of the brain were shown to nucleate local CD8^+^T cell responses to a neurotropic virus by expressing CCR7 ligands (65). As TLS-associated LTo-like cells are considered resident and long-lived, in comparison to the circulating and short-lived hematopoietic cells, they may hold the key to the reversibility of TLS assembly, providing an interesting therapeutic target for this process.

**Table 1:** **Markers associated with fibroblasts found in tertiary lymphoid structures observed in disease settings in mice and human**.

Disease	Mouse	Human	Reference
Sjögren’s syndrome (salivary glands and lacrimal glands) 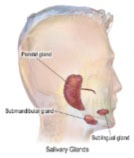	Podoplanin/gp38, CD21, CXCL13, CCL21, VCAM-1, ICAM-1, ER-TR7, FAP	Podoplanin/gp38, CD21, Collagen I, Laminin, CXCL13, CCL21, CXCL12, BAFF, VCAM-1, ICAM-1, ER-TR7, FAP	(63, 66–69)

Primary biliary cirrhosis, primary sclerosis cholangitis (liver) 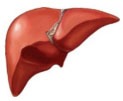	n.d.	Podoplanin/gp38, CD21, Collagen I, Laminin, CCL21, MadCAM-1	(63, 70, 71)

Rheumatoid arthritis (joints) 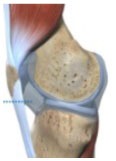	Podoplanin/gp38, VCAM-1, CXCL13, CCL21, FAP, Thy1.1, Cadherin-11	Podoplanin/gp38, VCAM-1, FAP, CD21, CXCL13, CCL21, RANKL	(61, 63, 72–75)

Atherosclerosis (arteries) 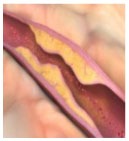	Podoplanin/gp38, VCAM-1, ER-TR7, LTβR, αSMA, CD35, CXCL13, CCL21	n.d.	(76–79)

Autoimmune encephalitis/multiple sclerosis (central nervous system) 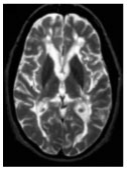	Podoplanin/gp38, PDGFRα, PDGFRβ, VCAM-1, ICAM-1, ER-TR7, Fibronectin, Thy1.1, Cadherin-11	CXCL13, BAFF	(64, 80)

Inflammatory bowel diseases (colon and small intestine) 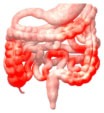	VCAM-1, ICAM-1, CXCL13, CCL21, CXCL12, CD21/35, Podoplanin/gp38, ER-TR7	Podoplanin/gp38, CXCL13, αSMA, FAP, CD21	(81–85)

Mucosal-associated lymphoid tissue (MALT) lymphoma 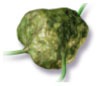	n.d.	Podoplanin/gp38, CXCL13, CCL21, CXCL12	(67, 86)

*Helicobacter pylori* gastritis (stomach) 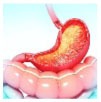	FDC-M1	CD21, CXCL13, CCL21	(86–88)

Inducible bronchus-associated lymphoid tissue (iBALT) (lungs) 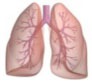	CD21/35, FDC-M1, CXCL13, CXCL12, CCL21, CCL19, CD90, Podoplanin/gp38	CD21, CXCL13, CCL21, CCL19, αSMA	(89–92)

Diabetes (pancreas) 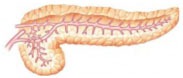	Podoplanin/gp38, FDC-M1, CXCL13, CCL19, CCL21, CXCL12, BAFF	n.d.	(63, 93, 94)

## Priming of Fibroblastic Stromal Cells at Sites of Inflammation

The cellular and molecular requirement for mesenchymal cell priming, leading to cell differentiation and specialization during TLS establishment, is debated. Transgenic expression of lymphoid tissue chemokines such as CCL21 and CXCL13 overcomes the requirement for LTi cells, but not of LTα1β2 in non–spontaneous models of TLS that form in the eye, thyroid, and pancreas (95–98). The absence of LTi cells, in a CXCL13 transgenic model, leads to the development of smaller and less organized infiltrates suggesting a specific role for those cells in developing larger and more complex infiltrates (63). However, because the aggregates in these models develop perinatally and in the absence of inflammation, this model cannot be considered a classical model of TLS, and conclusions on its elements should be carefully drawn.

The hierarchy of requirement of TNF family members in physiological lymphoneogenesis is clear, with LTα^−/−^ mice lacking both LTα3 and LTα1β2 expression showing the most severe phenotype, characterized by lack of all LNs and PPs, and a disorganized splenic white pulps (99, 100). In contrast, LTβ^−/−^ mice, which specifically lack LTα1β2 function, retain MLNs and cervical LNs, and their splenic defects are less pronounced than those of LTα^−/−^ mice (101, 102). A similar phenotype is observed in pregnant mice treated with LTβR–Ig fusion protein, whose progeny lack most PLNs and PP but retain MLNs (103–105). Ruddle and her group have clearly shown that ectopic expression of either TNFα, LTα, or LTα1β2 regulates the assembly of organized TLS, with the formation of MAdCAM^+^ (in LTα transgenic) and PNAd^+^ HEV (in LTα1β2 transgenic mice) and a complex network of lymphoid tissue chemokine expression (58, 106). In general, the combined expression of both LTα and β goes along with the formation of better organized lymphoid structures (58).

In spontaneous models of TLS formation, LTα1β2 is not absolutely required to prime the stromal cell compartment. Accordingly, the upregulation of adhesion molecules and the transient expression of lymphoid chemokines can occur in the absence of LTα1β2, or LTi and lymphocytes. Peduto and colleagues first demonstrated that a population of αSMA^+^ podoplanin^+^ fibroblasts, which express lymphoid chemokines and survival factors, classically associated with lymphoid stroma in SLOs, can differentiate in non-lymphoid tissue during inflammation and cancer. This phenomenon occurs prior to lymphocyte infiltration in the tissue and is conserved in RORγ-deficient mice (27). Other leukocytes, which are more abundant in the earliest phases of inflammation, such as myeloid cells or granulocytes, might therefore assume a “initiator role” in the formation of TLS by releasing proinflammatory cytokines capable of inducing activation of resident fibroblasts (27, 69, 107–109).

Since, by definition, TLS arise at sites of inflammation, it is virtually impossible to exclude the contribution of one or more of the TNF family members to the early phases of TLS formation. Engagement of TNFR on inflammatory or lymphoid tissue fibroblasts is known to upregulate chemokines, cytokines (including BAFF and IL-6), and adhesion molecules that largely define a primed stroma (110, 111). Moreover, TNF is known to upregulate the receptors for some of the inflammatory cytokines proposed to be involved in TLS establishment (112, 113). One may therefore put forward the hypothesis that the engagement of TNF is key prior to, or synergistically, with the expression of other proinflammatory cytokines to drive the initial priming of resident fibroblasts into functional LTo cells.

Other members of the TNF family have been implicated in lymphoneogenesis. Transgenic expression of LIGHT has been shown to induce TLS formation in models of melanoma and fibrosarcoma (114, 115) and to exacerbate disease in NOD mice (93). Overexpression of RANKL can also support the establishment of lymphoid tissue characterized by stromal cell production of lymphoid chemokines and lymphocyte recruitment (116–118). However, the mechanism by which RANKL regulates TLS assembly is not clear.

**Figure 1:**
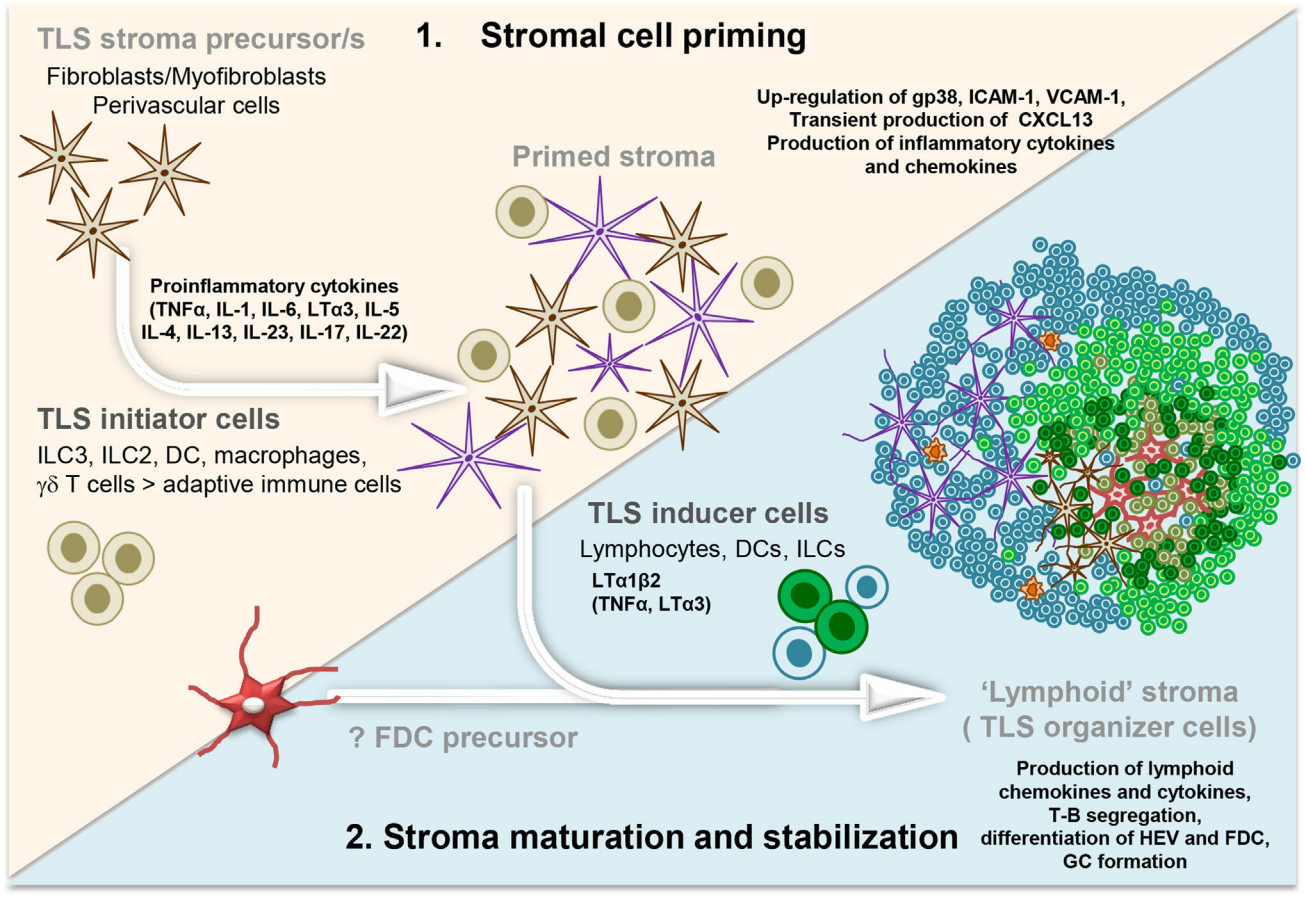
**Development and maturation of lymphoid tissue-like stromal fibroblasts in tertiary lymphoid structure (TLS)**. Multistep model illustrating the priming followed by the stabilization and maturation of the fibroblasts allowing a lymphoid tissue-like organization and function of the lymphocyte infiltrate. Acute inflammation within a tissue results in the localized production of several different proinflammatory cytokines by infiltrating leukocyte populations (initiators) and tissue-resident cells. These signals elicit “priming” of local stromal fibroblasts, which may include the upregulation of gp38, adhesion molecules, and inflammatory cytokines. Prolonged inflammation can lead to local production of LTα1β2, along with TNFα and LTα by hematopoietic inducer cells. This triggers changes in the stromal fibroblast phenotype and function, including the production of chemokines typically expressed in lymphoid organs. FDC differentiation from local fibroblasts might only occur at this stage, when a critical mass of LTα3/LTα1β2/TNFα signals are provided by the co-localizing B cells, possibly inducing a positive feedback loop. Organization of the resident stroma and hematopoietic cells in a T cell (blue cells) and B cell (green cells) rich zone enables priming and activation of T and B cells toward locally displayed antigens. The formation of GC supports affinity maturation and expansion of B cells clones and plasma cells. Not Illustrated: the presence and differentiation of the vascular network [reviewed in Ref. (57)].

Besides TNF and LT family members, the types of cytokines involved in the first phase of stromal cell priming in TLS vary according to the tissues and types of responses (Figure 1). IL-6 has been associated with the perivascular accumulation of B cells and mature plasma cells (119). In a model of subcutaneous tumor apoptosis, TGF-β has been demonstrated to induce CXCL13 expression (120), while IL-5 expression has been associated with iBALT development and lung disease (121). IL-4 and IL-13 are known to stimulate, to different extents, the upregulation of adhesion molecules and transient chemokine expression on fibroblasts (122). Finally, IL-4, IL-7, and to a lesser extent IL-15, are known to stimulate expression of LTα1β2 on naïve T lymphocytes that might lead to TLS formation [reviewed in Ref. (95, 123)].

A separate case needs to be made for cytokines belonging to the IL-23 family. IL-17 production by γδ T cells has been demonstrated to provide the trigger for priming of lung fibroblasts in iBALT (92). During EAE, the development of a PDGFRα^+^PDGFRβ^+^ podoplanin^+^CD31^−^ stromal cell network has been shown to be dependent on both IL-17 and IL-22 (64). Within murine salivary glands IL-22, but not IL-17, was deemed a key requirement for stromal cell activation in TLS that form (69). IL-23 expression has been associated with TLS formation in a model of arthritis (124), and the transfer of Th17 cells is sufficient to induce TLS during central nervous system tissue inflammation (109). Interestingly, the production of these cytokines and their relative source appears to be linked to the presence of the transcription factor RORγ that is also required for development and function of ILC3, γδ T cells, and Th17 cells (125–130). Several factors, like IL-23, may act upstream of RORγ, with IL-17 being downstream (131), thus increasing the level of complexity of this system. In some settings, RORγ is deemed dispensable, such as in virus-induced salivary gland infection, where TLS formation and autoantibody generation strongly depend on IL-22 but not IL-17 or RORγ (69). In a model of airway damage and inflammation, it has been shown that IL-17A might regulate both the expression and the proinflammatory properties of IL-22 (132), thus suggesting the possibility that several cytokines can initiate TLS establishment and their relative contribution is likely to be influenced by the site of inflammation and etiological agent.

To date, only one cytokine, IL-27, has been identified that directly inhibits TLS development by negatively regulating the differentiation of Th17 cells, a major driver of TLS development and RA pathogenesis (133).

In summary, multiple pathways and several cell types can act as initiators of TLS assembly and induce activation or priming of the resident fibroblasts in a way that leads to a lymphocyte permissive tissue state (Figure 1). The capacity of leukocytes, other than T and B cells, to provide cytokines for stromal cell activation demonstrates a critical uncoupling between stromal cell priming and lymphocyte accumulation in TLS, establishing a model whereby stromal cell priming might occur prior and largely independently from a significant lymphocyte migration into the tissue.

## Maturation and Stabilization of Fibroblastic Stromal Cells Allowing TLS Formation

Transient activation of stromal cells that often occurs in acute phases of inflammation is not sufficient to support complete lymphoid-like fibroblast maturation associated with TLS formation. Upon resolution of inflammation, the “primed state” of fibroblasts is likely to be lost; alternatively, these activated cells may disappear. Only selected circumstances, such as antigen persistence or severity and length of the inflammatory response, may drive the development of lymphoid tissue-like mesenchyme. Part of this complex phenomenon is also reliant on the dramatic changes that both stromal cells and leukocytes induce in the lymphatic and blood vasculature and that occurs as an integral part TLS development (57, 134). In this context, the ability of TLS-associated fibroblasts to secrete pro-angiogenic factors, including VEGF-C and VEGF-D, should also be highlighted (27).

Interestingly, this two-step process of mesenchymal cell priming and maturation is reminiscent of the early phases in the process of lymphoid neogenesis, whereby the early production of CXCL13 and the upregulation of ICAM-1 and VCAM-1 on local mesenchymal cells occur independently of lymphotoxin and LTi. In SLOs, this first phase is followed by the LTα1β2-dependent interaction of LTi cells with the primed mesenchymal cells leading to their specialization as LTo cells capable of inducing HEV development, lymphocyte recruitment, and stromal cell specialization in various subsets (28, 29, 135). Interestingly, in SLOs, the resident mesenchyme is unable to maintain the durable production of survival factors and chemokines if TNFR or LTβR engagement is missing and only a few disorganized LN form in LTα^−/−^ or LTβR^−/−^ mice (38, 50). Accordingly, the prolonged treatment of adult wild-type mice with LTβR-Fc leads to dedifferentiation of FDC, HEV, and partially fibroblastic reticular cells (FRC), and as a consequence to reduced lymphocyte recruitment, retention, and compartmentalization (136).

Similarly, in TLS, full differentiation of the lymphoid tissue-like fibroblasts also requires the presence of lymphocytes and LTβR signaling. TLS can form in LTα^−/−^ mice but display a disorganized pattern of lymphocyte aggregation, in absence of clear B/T cell segregation or HEV differentiation (90). LTβR-Fc treatment of established TLS leads to the same outcome, suggesting a continuous need for these signals in order to maintain differentiated HEV and chemokine-expressing fibroblast networks (95, 137, 138). Continuous LTβR and TNFR1 signaling is also required for sustained expression of VCAM-1, CXCL13, and CCL21 in TLS that form in the aorta and in the brain (64, 76, 77). This finding is reminiscent of the requirement for LTα2β1 in order to maintain normal numbers and compartmentalization of lymphocytes in the spleen, PP, and ILFs (47, 53, 137, 139, 140). The combined activation of TNFRI and LTβR is required for the formation of TLS in both inducible and spontaneous models of atherosclerosis (76, 77). However, blockade of LTβR is sufficient to reduce insulitis and diabetes (138) in a NOD mouse model characterized by the presence of podoplanin^+^ FRC networks and HEV differentiation (63).

All together, these data are consistent with a model of TLS formation in which there is an initial phase of stromal cell priming that occurs independently of LT and precedes tissue infiltration by adaptive immune cells. In the second step, the maturation of resident fibroblasts to a full LTo phenotype appears to be dependent in most settings on LT and TNF, presumably needed to enable dual activation of the NF-κB cascade, with the alternative pathway being maintained over time. Of note, continuous and strong expression of TNF and possibly other NF-kB activating cytokines may bypass this LTα1β2 requirement and still lead to TLS development, though the precise mechanism by which this phenomenon occurs has not been fully clarified. In the context of ectopic lymphoneogenesis, one may therefore propose an extension of the term “lymphoid tissue inducer” to different leukocyte cell types that express sufficient levels of LTα1β2 to induce full differentiation of resident mesenchymal cells into a lymphoid tissue phenotype (96, 108, 141–145). Naïve B cells and DC may qualify for this term, as these cell types on a wild type but not on a LT-deficient background can induce formation of lymphoid tissue structures *in vivo* (47, 146). Evidence is less strong for a LTi like role for T cells although they can express LTα1β2 upon cytokine exposure or activation (147).

Downstream the activation of LTβ receptor is the production of lymphoid tissue chemokines, critically required for lymphocyte recruitment and TLS development. Accordingly, ectopic CCL19 expression under the rat-insulin promoter alone is able to form small infiltrates rich in T cells and dendritic cells; while ectopic CCL21 expression is able to induce the formation of large and better organized infiltrates, characterized by the specific development of T and B zone stroma and HEV differentiation. Similarly, ectopic CXCL13 expression is known to regulate lymphoid tissue neogenesis with T and B cell segregation and complete specialization of the stromal compartment, including FDCs and HEVs (63, 137). These findings in TLS formation are in keeping with the critical role played by CXCL13 and CCL21 in lymphoid organ development during embryogenesis (95).

Interestingly, different functional phenotypes, in terms of chemokine expression, have been observed in fibroblasts isolated from different anatomical sites. In the skin, podoplanin^+^ inflamed fibroblasts express modest levels of IL-7 and CXCL13, but high quantities of CXCL12 and VEGF-C (27). In contrast, podoplanin^+^ cells isolated from gut and tumors significantly upregulate the lymphoid chemokines CXCL13, CCL19, CXCL12, and various cytokines, including VEGF-C, connective tissue growth factors, including fibroblast growth factors (27). Stromal cells isolated from iBALT are characterized by CXCL12 expression (92), while the network of podoplanin^+^ CD31^−^ cells isolated from salivary gland TLS express CXCL13 and CCL19 but not CXCL12 (69). Differences in terms of TLS organization and chemokine expression can also be observed at different sites or even in TLS at the same anatomical site when they are induced in response to different antigens. For example, intranasal administration of the poxvirus modified vaccinia virus Ankara (MVA) in mice is able to induce highly organized iBALT with B cell follicles containing a network of CXCL13-expressing FDCs and CXCL12-producing follicular stromal cells. However, mice treated with *P. aeruginosa* developed iBALT with B cell follicles that consisted of CXCL12^+^ follicular stromal cells but not CXCL13^+^ FDC (92). The signals responsible for these phenotypic and functional differences are unclear and most likely result from an integrated response to different anatomical environments, antigenic stimulations, and inflammatory milieus. While this review focuses on fibroblasts, the ability of hematopoietic cells, including macrophages, dendritic cells, and Th17 cells, to ectopically express CXCL13 should also be acknowledged, suggesting the possibility to consider the cytokine/chemokine, rather than its cellular origin as a therapeutic target (148–152).

## Stromal Fibroblast Organization in SLOs and TLSs

Mature SLOs are characterized by the anatomical organization of lymphocytes in distinct compartments, which is due to the segregated chemokine expression of CCL19/21 in T zones and CXCL13 in B zones. The source of these chemokines are specialized subsets of resident FRC and FDC, which attract and retain specific leukocyte populations, besides providing survival factors.

**Figure 2:**
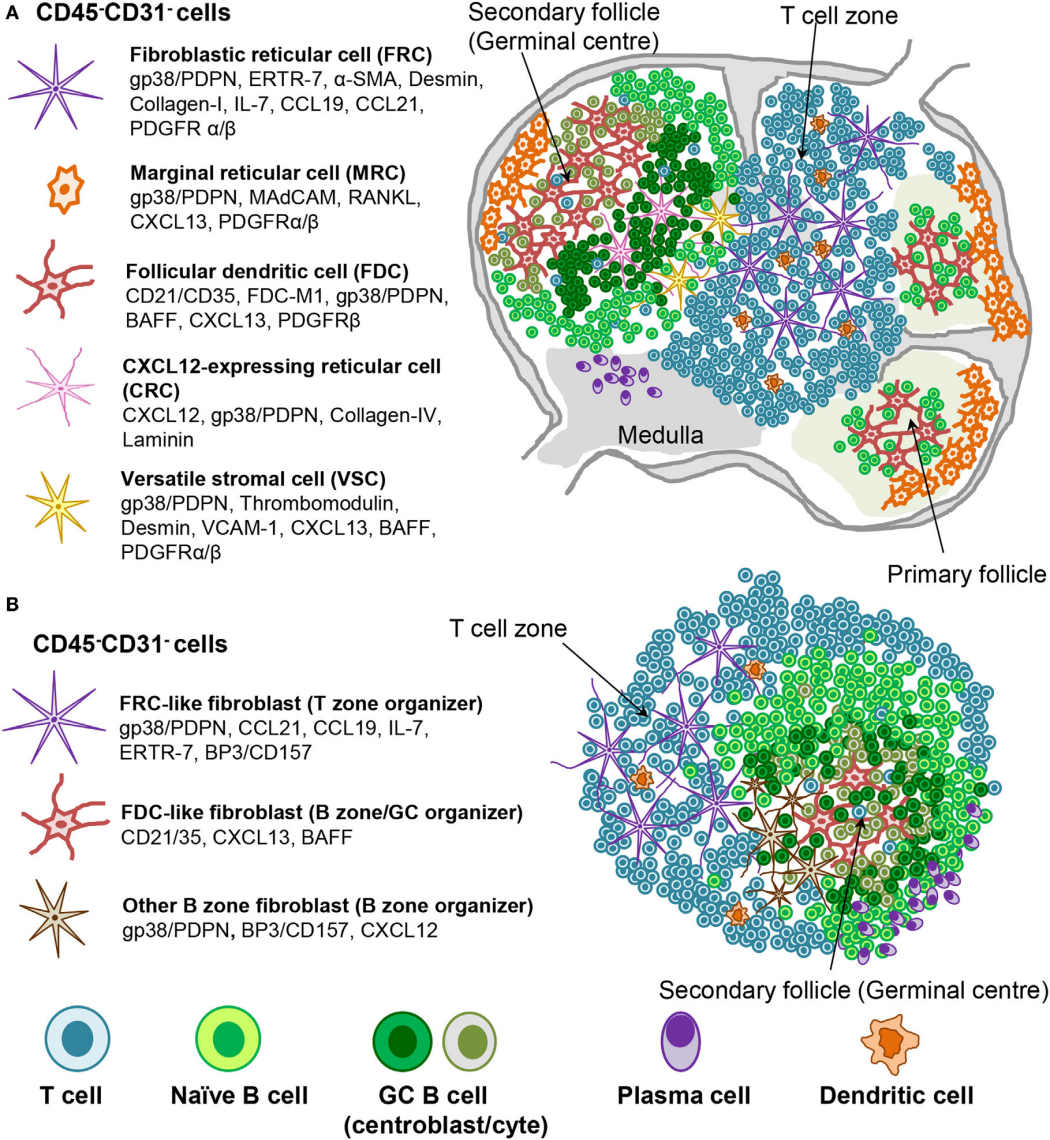
**Stromal fibroblast populations in SLO and TLS**. **(A)** Fibroblast populations in the lymph node control the organization and survival of lymphocytes in distinct areas. Fibroblastic reticular cells (FRC) produce CCL19 and CCL21 along with the survival factor IL-7 to attract and maintain T cell populations and provide a niche in which their interaction with dendritic cells (DC) can occur. Follicular dendritic cells (FDC) produce CXCL13, which attracts CXCR5^+^ cells to the B cell follicles. Other stromal cells are thought to play a role during the germinal center reaction (i.e., CRCs) or in antigen delivery (MRC). **(B)** Mature TLS are characterized by segregation into distinct T cell and B cell areas including the presence of germinal center like structures and areas rich in plasma cells. Stromal cell populations that perform comparable functions to those found within SLO can be identified, which underlie this T/B cell segregation within the larger TLS.

Fibroblastic reticular cell subsets, including FDC, are defined by their phenotype, anatomical location, and function (2, 24, 153, 154) (Figure 2). T zone FRCs or TRC classically inhabit the T cell cortex in LNs and are characterized by the expression of podoplanin and lack of the vascular marker CD31. TRCs are responsible for the recruitment, retention, and movement of naïve T lymphocytes and DCs *via* their expression of CCL19 and CCL21. Besides regulating immune cell trafficking, TRCs produce extracellular matrix, forming a system of microchannels (conduits) that connect the subcapsular sinus with the paracortex and HEVs. TRCs also provide a significant source of IL-7, which in combination with CCL19, sustains naïve T cell survival within the LN T zone, regulating T cell homeostasis (22, 155).

Fibroblastic reticular cells that inhabit the outer B zone, also termed B cell zone FRCs (BRC), are characterized by high levels of BP-3 and podoplanin expression but lack FDC markers like CD21/35. BRC represent an important source of oxysterol chemoattractants, BAFF, CXCL13, and the Notch ligand DL4 (156–158). Marginal reticular cells (MRCs) represent another B zone FRC population that sits underneath the subcapsular sinus and is characterized by RANKL and CXCL13 expression (35, 159). BRC and possibly MRC form conduit networks able to deliver lymph-drained information, including antigens, from the subcapsular sinus to the B cell area and eventually to the FDCs present within the B cell follicles (2, 153, 156–162). Due to their phenotypic similarities, MRCs were proposed to represent the adult equivalent of embryonic LTo cells (159). In support of this concept, MRCs were shown to be the precursors of LN FDCs during immune reactions (162).

Follicular dendritic cells are the prominent stromal cell subset found within the central part of primary B cell follicles and localize specifically to the light zone of the GCs in secondary follicles. FDCs are defined by their dendritic appearance and their capacity to retain opsonized antigen *via* Fc and complement receptors. FDCs serve as long-term reservoir of native antigen for the positive selection of affinity matured GC B cells or centrocytes (163). In addition, FDCs are a source of CXCL13 and BAFF, responsible for migration and survival of naïve B cells. TNFR and LTβR signaling are critically required for the maintenance of the FDC network and in general to support the CXCL13 producing stroma in the follicles (53, 164, 165), as evidenced by their rapid disappearance upon TNFR or LT blockade (45, 166–169).

Recently, a population of CXCL12-expressing reticular cells (CRCs) has been described in the dark zone of the primary follicles and polarized GCs, where they are required for the recruitment of CXCR4^+^ centroblasts and effective GC responses (170). This subset is fate mapped by both the *Cd21-cre* and *Ccl19-cre* mouse lines with the fate mapping indicating past and/or present expression (171). It lacks most of the classical markers of FDCs [such as CD21/35, VCAM-1, FDC-M2, FDC-M1, M-2, and CD16/32 (FcγRII/III)] or FRCs (such as laminin and type IV collagen association). Moreover, CXCL12 expression on CRCs is independent of LT or TNFα thus establishing critical phenotypic and developmental differences between CRC and FDCs (171). Finally, a population of versatile stromal cells (VSC) has been described at the T zone edges of the B cells follicle, able to respond to inflammatory stimuli, B cell contact, and LTα1β2 stimulation by upregulating CXCL13 expression (172).

Many of the FRC subtypes are fate mapped by the same *Ccl19-Cre* transgenic mouse, suggesting a common precursor in embryonic life for the majority of FRC subsets, at least in the LN (171). In future, mice allowing inducible fate mapping are needed to gather more direct evidence for such a precursor–progeny relationship, both in embryonic and postnatal LN. Currently, very little is known on the specific differentiation and survival signals leading to the complex stromal cell organization observed in adult life.

To study signals regulating FRC function, various FRC lines have been generated from adult murine and human SLO. Murine cell lines constitutively produce CXCL12 and sometimes BAFF but require stimulation of the TNF receptor to upregulate various inflammatory chemokines (e.g., CXCL10, CCL4/5) as well as IL-7 (33, 35). Stimulation of LTβ- or TNF-receptors strongly upregulates matrix production, with the combination of both signals showing synergistic effects. Those signals could be mimicked by CD4^+^ T cells, physiological neighbors of FRC *in vivo*. Similarly, stimulation of a MAdCAM^+^ MRC line *via* both LTβ- and TNF-receptors was also able to induce further expression of CXCL13 and CCL19 (33, 35). Interestingly, fluid flow was shown able to induce CCL21 expression in a FRC line (173); while human LN FRC responded to TNFα, IL-6, IL-4, and IL-13 by upregulating various cytokines, adhesion molecules, and metalloproteinases (174). These *in vitro* data support an ability of lymphoid tissue fibroblasts to adopt different functions depending on the signals received from the surrounding environment, and thereby influence neighboring immune or stromal cells.

Our knowledge about stromal fibroblasts within murine and human TLS is still very limited. Current evidence suggests the presence of TRC-like cells within the T cell rich zones, and of FDC-like cells that inhabit B cell rich areas in more organized TLS. MRCs, BRCs, CRCs, or VSCs have not yet been described in TLS. Depending on the size and type of TLS, fibroblasts display a variable degree of the phenotypic and functional features described for their SLO counterparts (Figure 2). It is debatable the extent to which TLS stroma reaches a comparable level of polarization and differentiation compared to SLO stroma. The two subsets identified so far in TLS, namely CCL21^+^ TRC and CXCL13^+^ FDC, can associate and probably form distinct compartments, such as T and B zones but also allow the formation of functional conduits and GCs (63). These niches appear to be functional in generating a specific adaptive immune response, but it is less clear whether fibroblasts in TLS allow a comparable regulation of immune processes. It is likely that in TLS, similar to SLOs, the predominant lymphocytic population that accumulate in each area imprint locally activated mesenchyme to support an increased requirement for specific survival and chemo-attractive factors. Lymphoid stromal cell differentiation would therefore be programed by anatomical location through contact with neighboring cells. Any polarization toward an FRC-like or FDC-like phenotype may be reenforced as lymphocyte segregation arises within mature TLS by the expression of one or two chemokines, such as CXCL13 and CCL21 (61, 66, 67, 72), thereby restricting stromal cell contact to either T or B cells. It is known that both CXCL13 and CCL21 can induce LTα1β2 expression in the responding lymphocytes, further enhancing chemokine expression by the neighboring fibroblasts (95, 142). The additional factors needed besides LTα1β2 to drive fibroblasts into a differentiation program specific for the B zone or the T zone are currently unknown.

In murine TLS, TRC-like cells expressing podoplanin and other markers (Figure 2; Table 1) are found throughout T cell rich zones as three-dimensional reticular networks and partly co-localize with dendritic cells thereby forming an environment where cognate T cell stimulation is possible. They associate with matrix fibers that can form functional conduits and connect with HEVs. Often they express CCL21, which is key to the T zone formation. CCL19 and IL-7 expressed by FRC may contribute to local T cell accumulation and possibly to random T cell migration along the TRC network (2, 153, 155, 175).

Follicular dendritic cell networks form only within B zones of large TLSs in mice and are then associated with functional GCs allowing B cell differentiation to affinity matured plasma cells (63, 88, 92, 94, 176, 177). Interestingly, not all tissues, and only a minority of TLSs form fully mature FDC networks, suggesting that while the stimulus for FDC differentiation might be the same as that observed in SLO (TNFα and LT), the threshold required for full FDC differentiation in peripheral tissue is higher. An alternative possibility is that FDC precursors in the periphery are scarce and differentially distributed in different organs. Whether the phenotype, origin, and function of FDCs in peripheral TLSs differ from those of SLO is not known, neither is their differential dependency on LT or LT-inducing pathways. It is important to highlight that even in SLOs, at least two different precursors have been identified for FDCs, which differ between spleen and LNs (162, 178, 179), thus potentially increasing the complexity of signals and progenitors required for FDC differentiation in TLS.

While it is likely that in TLS a single lymphoid tissue-like progenitor gives rise to both FRC and FDC-like cells, it cannot be excluded that FDC might differentiate from FRC-like cells or another precursor later in TLS development. Interestingly, the absence of MRC-like cells and the lack of any anatomical capsule within the TLS appears to exclude the involvement of an “MRC-like progenitor” in FDC differentiation in TLS. Data obtained from parabiosis experiments and fate mapping experiments by Peduto and colleagues suggest that local, rather than circulating, precursors are responsible for the expansion of the stromal network responsible for TLS establishment (27). Proliferation of the resident stroma has been also observed in an inducible model of TLS, both in the vascular and stromal compartments (134) (Nayar et al., manuscript in preparation), which mimics the expansion of the stromal compartment during immunization in SLOs (22, 180–182). Therefore, the most likely scenario is that local differentiation and expansion of tissue-resident mesenchymal cell/s accounts for TLS development.

Even less is known about fibroblasts found in TLS within human pathology, in part due to the lack of markers identifying fibroblasts and distinguishing different cell subsets. Most efforts have concentrated on reporting the presence or absence of CD21^+^ FDCs or lymphoid tissue chemokines, such as CXCL13, CCL21, CCL19, and CXCL12 (7, 8, 183). Discrete expression of CXCL13, CXCL12, and CCL21 has been described in salivary glands of patients with SS, RA, multiple sclerosis, primary sclerosing cholangitis, atherosclerosis, inflammatory bowel disease, chronic lung diseases, *Helicobacter pylori*-induced gastritis, and lymphoma [reviewed in Ref. (7)]. However, this classical work largely preceded the phenotypic characterization of fibroblast subsets in mice and therefore lacks in-depth characterization of the stromal cell compartment. Nonetheless, the association between the level of organization of TLS with lymphoid chemokine expression (7), together with identification of the source of lymphoid chemokines in the αSMA^+^ or desmin^+^ stromal compartment (63), suggests that TLS fibroblast subsets exist in humans. More recently, our laboratories have demonstrated the presence of podoplanin^+^ FRC-like cells in tonsil, RA synovium, and SS salivary glands, as based on the expression of podoplanin and CCL21 and the association with T cells, DC, and matrix fibers (63). These FRC-like cells were distinct from the CD21^+^ FDC that organized B cell rich zones, as described previously by several laboratories and summarized in Table 1.

While the use of podoplanin to identify “lymphoid tissue stromal cells” in the periphery remains a reasonable approach, podoplanin expression has also been observed on epithelium, lymphatic vessels, Th17 cells, and myeloid cells (184). Its use therefore requires careful analysis of cell morphology and double labeling with cellular markers (absent on fibroblasts) to validate the specificity of the population of cells detected. In future, it will be important to use further markers to discriminate human fibroblast subsets in TLS, in order to improve stromal cell characterization in human TLS histology samples.

## Effector Functions of TLS

Tertiary lymphoid structures are classically defined as lymphoid aggregates forming in organs whose main function is other than the initiation of an adaptive immune response. TLS appear to form there because of an abundance of antigen, either “self” in autoimmune diseases, “self” or “altered self” in cancer, or foreign antigen/s during infections and transplant rejection. At those ectopic sites, TLS can contribute to the generation of an antigen-specific immune response with plasma cell and antibody generation, often maintained by the persistence of the antigen and/or inflammatory signals (6, 8). The local stromal structures needed for naïve cell recruitment and affinity maturation of the B cell compartment are mainly observed in the most organized TLS (66, 185). Interestingly, TLS do not develop in all forms of chronic inflammation and only arise in certain permissive tissues. A classical association with mucosal epithelium has been observed. However, TLS can form in the synovium, a tissue devoid of epithelial structures (7). The factors involved in tissue permissiveness are not clear, and while it is intuitive to suggest the need for proximity to antigen and antigen presenting cells, this is not sufficient as exemplified by the case of the skin, classically considered a “hostile site” for TLS formation.

Aggregation of lymphocytes in small TLS is commonly observed in transient infections where it is considered a positive development, aimed at containing local infections. The development of lung TLS in response to influenza supports the further development of a strong antigen-driven T cell response contributing to viral clearance (186, 187). In these cases, TLS disappear shortly after pathogen clearance leaving the tissue intact (68, 188).

However, in chronic inflammation, the presence of TLS has been associated with poor clinical outcome and disease progression rather than resolution (94, 189). TLS formation correlates with serum autoantibody levels, disease severity, tissue damage, and decreased organ function in several diseases including SS [reviewed in Ref. (7)]. In RA, the formation of subchondral bone TLS supports osteoclast activation and tissue damage (190), and the presence of synovial TLS associates with anti citrullinated antibody production and poor response to anti-TNF antibodies (74, 191). Accordingly, levels of TLS-associated CXCL13 expression correlates with disease severity and persistence of subclinical synovitis (190, 192, 193).

It is arguable that TLS represent a response to and are not *per se* a cause of inflammation. However, a combination of factors, among which excessive cell recruitment, poor lymphatic drainage, disorganized cellular interaction, and excessive survival factors can contribute to TLS persistence in tissue, favoring a pathogenic role in the context of diseases. Interestingly, not all patients with autoimmune disease develop TLS, despite the presence of factors associated with its development, for example, Th17 cells in RA (133). This suggests that the biological relationship between disease progression, clinical features, and TLS formation is more complex. In this context, the detection of GC within salivary glands of patients with SS has been classically associated with increased risk of lymphoma (194), suggesting that chronic antigen stimulation and excess of survival factors might favor the development of malignant B cell clones. However, this proposal is controversial and a strong positive correlation between the two pathogenic entities cannot be identified, leaving the relationship between TLS formation and lymphoma development uncertain (194, 195).

The role of TLS in the context of cancerous growth is also debated. TLS are believed to sustain the antitumor response in solid malignancies that arise in the colon, breast and ovaries as the presence of TLS in the context of cancer has been associated with a favorable prognosis (196). Nonetheless the ability of tumor cells to induce T regulatory cells (T_reg_) and suppress the host immune response is well known and there is the possibility that cancer cells highjack TLS to exert this immunosuppressive function (197–200).

Classically, the key effector function associated with TLS development has been the formation of GCs and the production of autoantibodies [reviewed in Ref. (7)]. However, more recently a more complex role for TLS in the context of T cell activation and maturation of pathogenic T cell response has been proposed.

While the production of CXCL13 represents a sensitive and powerful readout of stromal cell activation in TLS (69), this is unlikely, in the early phases of TLS establishment, to be restricted to the rather complex events of follicular B cell differentiation, an event that occurs later in TLS (69). It is likely that early CXCL13 production in the context of TLS assembly is aimed at driving the recruitment of CXCR5^+^ B cells and T follicular helper (T_fh_) cells (201). Accordingly, interfering with T_fh_ infiltration by ICOS-L blockade results in reduced TLS assembly and progression of vascular disease in a model of TLS associated with atherosclerosis (202). The origin of T_fh_ that arise within TLS is currently not clear. As mentioned, T_fh_ populations have been identified within the circulation in both mice and humans that could be recruited into TLS by newly established CXCL13 gradients (203–205). There is however the possibility that TLS provide a site for local T_fh_ differentiation. In support to this hypothesis, naïve T cell recruitment and priming has been reported within TLS that form in pancreatic tissue in NOD mice (93). However, naïve T cell recruitment requires the upregulation of CCR7 and L-selectin ligands on the vascular endothelium, typically HEVs (59, 206), which is another hallmark of mature TLS. Effector T cells are more likely to be recruited in the earliest phases of TLS assembly, thus suggesting that in TLS, as opposed to SLO, T cell differentiation into T_fh_ might occur from previously activated peripheral T cell populations that already display effector functions. In support of this hypothesis, Th17 cells isolated from TLS of EAE affected mice display features of T_fh_, including the upregulation of expression of CXCR5, ICOS and Bcl6 (109). Within TLS the activated fibroblast compartment may provide key signals for T cell differentiation, such as IL-6, required for the induction and maintenance of the T_fh_ phenotype (207, 208), thus suggesting an additional role for stromal cells in the context of TLS development.

The presence and function of T_reg_ populations in the context of TLS has been less studied. T_reg_ recruitment into TLS might directly interfere with the activity of TLS associated T_fh_ cells, similarly to what has been described in SLOs (209, 210). Accordingly, in TLS that form in association with atherosclerosis, disruption of CD8^+^ T_reg_ activity is known to induce an expanded GC B cell response (202). Additionally, in a mouse model of lung adenocarcinoma, T_reg_ that inhabit local TLS, are known to interfere with the antitumor T cell response. In addition to directly inhibiting T cell responses within the TLS, T_reg_ appear to impact upon the recruitment of additional lymphocytes to the TLS by affecting the formation or maintenance of PNAd^+^ HEVs (211, 212). Interestingly, the tumor microenvironment is conducive to the recruitment, generation, and maintenance of T_reg_ populations (197) and, indeed, T_reg_ infiltration in solid tumors is considered a negative prognostic factor. Whether T_reg_ are less active or abundant in TLS associated with chronic inflammation, as compared to cancer, remains to be answered.

T regulatory and Th17 cell differentiation is promoted by TGF-β; however, Th17 development occurs on a background of proinflammatory cytokines, such IL-6, IL-21, and IL-23 (213–215). It is possible that in TLS that arise in chronic autoimmunity, the inflammatory milieu, partly established by locally activated fibroblasts, positively enforce Th17 over T_reg_ differentiation, favoring the upregulation of the transcription factor RORγ while inhibiting FoxP3 expression (216). Additionally, an environment that is rich in IL-1 and IL-6 and deficient in TGFβ is sufficient to reprogram T_reg_ toward a Th17 phenotype (217, 218), thus suggesting that the inflammatory microenvironment associated with TLS in chronic inflammation may not be able to maintain T_reg_ phenotype cells. Indeed, Th17 differentiation has been observed in RA TLS (133). The differences between diverse T effector populations and the balance between T effector and T_reg_ cells in the different lymphoid niches may therefore explain some of the discrepancies observed between immune responses that occur within TLS associated with cancer and chronic inflammation.

It is not clear to what extent the stromal cell compartment contributes to TLS pathogenicity. In SLOs, FRC are increasingly recognized as active modulators of the immune response. FRCs can interfere with the T cell response through several mechanisms, releasing soluble modulators that negatively regulate T cell proliferation or providing negative co-stimulatory molecules such as PD-L1 (219–221). The ability of FRC to acquire peptide–major histocompatibility (MHC) II complexes from professional antigen presenting cells or to upregulate MHC I and II molecules has also been shown. Moreover, FRC are able to display peripheral tissue antigens mediating the deletion of peripheral autoreactive CD8^+^ T cells (19, 222, 223) and maintaining the homeostasis of T_reg_ cells (222, 224). Taken together, these studies demonstrate that, while FRCs provide survival niches for lymphocyte homeostasis, they also govern the magnitude of the immune response (19, 221).

The extent to which the regulation of immune responses by stromal cell populations translates from SLOs to TLS has not been explored. Interestingly, fibroblasts isolated from non-inflamed peripheral tissues display a strong propensity to inhibit T cell responses, possibly to protect non-lymphoid tissues from the harmful effects of inflammation (221). In cancer-associated TLS, also characterized by a chronic inflammatory response, stromal fibroblasts are believed to contribute to immune evasion, preventing lymphocyte effector functions and immune cell access to the cancer site (12). However, in contrast with these, most recent findings and the immunosuppressive role described for stromal cells in SLOs and cancer, current evidence strongly suggests a pathogenic role for TLS in autoimmune conditions, sustaining lymphocyte survival and supporting lymphocyte persistence in the tissue (7, 8). The pathogenic, non-immunosuppressive role of lymphoid-like fibroblasts that inhabit TLS found in chronic inflammatory conditions appears therefore unique and requires further characterization. There is the possibility that functional differences exist among fibroblasts that bear a similar phenotype in TLS and SLOs or that functional differences are acquired during disease progression. The different origin of SLO and TLS fibroblasts and the cytokine milieu driving mesenchymal differentiation at ectopic peripheral sites are likely to contribute to this complex phenomenon. A more detailed characterization of the stromal compartment in TLS associated with chronic inflammation in comparison with SLOs might provide key elements to unravel these discrepancies.

## Stromal Fibroblast Deletion: A Novel Strategy to Manipulate the Immune Response

Interesting insights into the function of SLO fibroblasts have been derived from cell deletion experiments. Taking advantage of the mouse lines expressing diphtheria toxin receptor (DTR) selectively in fibroblasts by the use of the fibroblast-specific promoters of FAP (fibroblast activation protein α) or CCL19, stromal cell deletion could be obtained in adult LNs. *FAP–DTR* mice treated with DTX showed disrupted LN homeostasis with strongly reduced numbers of T and B lymphocytes and DCs. Upon influenza infection, mice lacking FAP^+^ cells mounted a diminished immune response characterized by reduced numbers of GC B cells, plasma B cells, and T_fh_ cells (225). By using a different inducible transgenic model [*Ccl19-*Cre × *Rosa26*-diphtheria toxin receptor (iDTR) mice] Turley, Ludewig, and colleagues demonstrated that selective depletion of FRCs resulted in aberrant localization of T lymphocytes within the LN cortex as well as a reduction in both CD4 and CD8 T cell numbers *via* a mechanism dependent on IL-7. Antigen-specific T cells isolated from these immunized, FRC depleted mice failed to undergo priming and proliferation (157, 226). Humoral responses and B cell homeostasis were also impaired in the absence of FRC, with disorganized B cell accumulation within the GCs and significant reduction of virus-specific antibody production (157). While this defect was largely attributed to the inability of B cells to access homeostatic levels of BAFF once FRC were depleted (157), Acton et al. recently demonstrated that the engagement of CLEC-2 (expressed on DC) by its ligand podoplanin (expressed on FRC) is necessary for DCs to spread, migrate, and provide appropriate Ag presentation to T cells in LNs (227, 228). This suggests that the inability of the DCs to migrate into LNs in FRC-depleted LNs could contribute to the defect in T and B activation observed in this model. It has been reported that ablation of FDC achieved in *Cd21-Cre* × *Rosa26-iDTR* mice results in loss of primary B cell follicles. This effect, partially mediated by BAFF and CXCL13 depletion, is also supported by the decreased levels of IL-6 and integrins present in SLO of transgenic mice treated with DTX (229). All together, these studies indicate that alterations in SLO stromal compartments can alter lymphocyte survival, compartmentalization, and immunological competency, often sequentially linked, which profoundly impact on SLO function.

The impact of stromal cell deletion on TLS has not been addressed as yet and might provide critical clues on the relative immunosuppressive or proinflammatory role of TLS-associated stromal cells, ultimately unveiling whether the role of TLS in different diseases at various anatomic sites is beneficial or detrimental. Whether this approach, using antibody-based therapeutic agents will be feasible in humans is of interest. Deletion of specific subsets of fibroblasts might be problematic due to the anatomical impact related to the apparent overlap of markers between lymphoid stroma in SLOs, TLSs, and non-lymphoid tissues. However, a series of compounds able to interfere with stromal cell activation and functions are available and will present an interesting avenue to target stromal cell activation, alone or in combination with current immunomodulatory agents, such as anti-TNFα, anti-IL-6, anti-CTLA4 and anti-PDL1.

## Conclusion

The main function of TLS is to maintain lymphocytes populations and provide a level of structural organization that enables the development and regulation of an adaptive immune response within peripheral tissues. This role is largely played by distinct populations of activated mesenchymal cells that acquire features similar to those of the lymphoid fibroblasts that inhabit SLOs. However, fibroblast maturation in TLS is an event, which is highly influenced by the anatomical site, the danger signals, and the inflammatory microenvironment, thus profoundly differing from the stereotypic mesenchyme specification observed during SLO development. Moreover, the organization and size of TLS does not equal that of their highly regulated SLO counterparts. The likely inability to precisely control leukocyte recirculation and cross talk within diseased tissue together with the absence of finely regulated chemokine gradients for lymphocyte positioning and the abundance of lymphocyte survival factors might explain why TLS fail to resolve and thereby contribute to immune-mediated inflammatory disease and its persistence.

## Author Contributions

All the authors contributed in writing the review.

## Conflict of Interest Statement

The authors declare that the research was conducted in the absence of any commercial or financial relationships that could be construed as a potential conflict of interest.
